# An Exploratory Study on Corporate Governance From Neuro-Governance Lenses in the Malaysian Context

**DOI:** 10.3389/fpsyg.2022.911907

**Published:** 2022-06-15

**Authors:** Larisa Ivascu, Codruta Daniela Pavel, Muddassar Sarfraz, Benedict Valentine Arulanandam, Hong Yip Tan

**Affiliations:** ^1^Faculty of Management in Production and Transportation, Politehnica University of Timişoara, Timişoara, Romania; ^2^Academy of Romanian Scientists, Bucharest, Romania; ^3^Faculty of Economics and Business Administration, West University of Timişoara, Timişoara, Romania; ^4^School of Management, Zhejiang Shuren University, Hangzhou, China; ^5^Sunway College, Kuala Lumpur, Malaysia; ^6^Independent Researcher, Kuala Lumpur, Malaysia

**Keywords:** psychology, neuro-governance, neuro-accounting, neuroeconomics, neuro-ethics, human nature

## Abstract

Our minds are powerful, creative, forceful, and strong, controlling our thinking and behaviors. A series of high-profile accounting and financial scandals have been revealed in the past few decades, and the Enron case was the most representative of them all. Corporate decision-makers have traditionally enjoyed high remunerations, compensations, and social status. Hence, the underlying rationales and motivation drivers that motivate managers to conduct unethical behaviors have always been a heightened concern. This research aims to delineate the narratives of corporate governance misconducts and the underlying rationales of these unethical behaviors. This study incorporates independent variables of neuro-accounting, neuroeconomics, neuro-ethics, and human nature using a qualitative methodology. From this study, the social norm of fairness showed that the human nature of greed and selfishness would motivate corporate decision-makers to engage in any exchange that could benefit themselves, although it is unethical and illegal. Second, neuroeconomics revealed that scarcity of economic resources, level of risks and uncertainties, and expected rewards could be the factors that motivate managers to conduct unethical behaviors, especially when their remunerations are tightly linked to company performances. Third, neuro-ethics shows that managers who lack moral values, have unstable emotions, and possess negative moral intuitions or personal assumptions could be more likely to pursue their interests at the cost of others. Lastly, neuro-governance also proved that self-benefits and financial incentives will usually be the priority and would be a motivating factor for misconduct.

## Introduction

Over the past several decades, corporate governance has often come under the attention of investors, government regulators, professional bodies, and researchers (Hamza, 2018). The Asian Financial Crisis in 1997 caused the global economy to collapse, especially in countries such as South East Asia, Korea, and Japan (Saltaji, 2018). It caused a series of high-profile accounting and financial scandals, fraudulent activities, and deliberate manipulations of financial statements, which led to the collapse of corporations such as Enron, WorldCom, and Satyam Computers Limited (Prusa, 2016; Al-Dhamari et al., 2018; Masli, 2018).

In the aftermath of the events mentioned above, the need for corporate governance mirrored an increased tendency in many developed and emerging economies (Saltaji, 2018). The Congress of the United States passed the Sarbanes Oxley Act in 2002, while the Malaysian government also implemented the Malaysia Code of Corporate Governance in March of 2000 (Othman et al., 2013). However, these remedies were overly stressed on corporate check and balance regime facilitation. They seemed to overlook the origins of the corporate scandals: human nature of greed and self-interest. Therefore, the need to study human nature, decision-making, and behaviors is rudimentary and of heightened concern. Recent studies have emphasized the influence of artificial neural networks in decision-making but do not touch on the fundamentals of neuro-accounting, neuro-ethics, neuroeconomics, and human nature (Turluev and Hadjieva, 2021).

**FIGURE 1:**
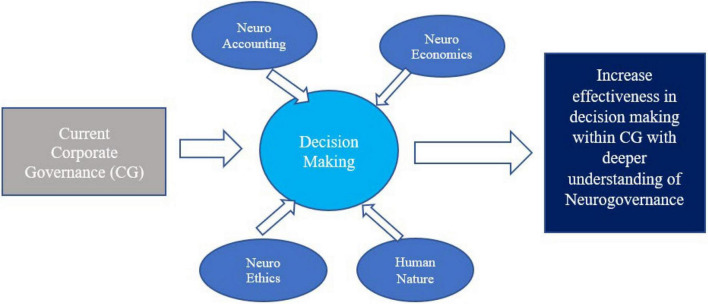
Theoretical framework denotes the components that lead to neuro-governance (Source: Authors own).

Nevertheless, Patterson and Pardo (2016) stated that psychology disciplines alone seem to be insufficient in human decision-making, and that behaviors are complicated and sophisticated. Therefore, as there are limited studies within the Malaysian context, this research has specifically selected the neuro-governance discipline to enhance the understanding of human agency, mental states, mindsets, and the underlying rationales of corporate unethical behaviors in the Malaysian corporate arena. This research aims to provide in-depth reviews on human decision-making and behaviors and the underlying rationales of corporate misconduct from views of neuro-accounting, neuroeconomics, neuro-ethics, and human nature (see Figure 1).

This research addresses the following questions: (1) is effective corporate governance structure important, (2) does neuro-governances provide significant explanations and examinations on how humans think, perceive, judge, and behave, and (3) is truly understanding human behaviors and decision-making important to mitigate corporate unethical behaviors and misconducts, and, therefore, enhance corporate governance functions?

## Literature Review

### Corporate Governance Concept

The term governance originates from the Latin word *gubernare*, meaning to steer, and this word generally applies to the ship’s steering. Therefore, corporate governance implies providing future direction for a company to achieve its desired goals and objectives rather than direct control (Dibra, 2016).

There are no uniform definitions for corporate governance in the world. For instance, the Organization for Economic Cooperation and Development (Organisation for Economic Co-operation and Development, 1999) defines corporate governance as the relationships between participants of the governance structure, such as shareholders and stakeholders, and how a company is managed. The Berlin Initiative Code and Federation of Belgian Corporation described corporate governance as a set of legal regulatory frameworks to direct and manage a company (Borgia, 2005).

Corporate governance is the most critical pillar in the structure of a company, which concerns facilitating an effective internal monitoring and controlling mechanism for the company’s day-to-day activities and operations. Specifically, corporate governance has concerns about the company’s internal control system, risk management, auditing, and integrity management to ensure the company is well-managed by optimal internal risk elimination. Furthermore, corporate governance is specifically concerned with strategy-making (Othman et al., 2013). Major shareholders generally would be the primary party that rules the company. Hence, the corporate governance system is aimed to prevent any individual, including major shareholders, from having too much influence and power to protect the interest of shareholders and stakeholders (Saltaji, 2018).

Moreover, contemporary corporate governance aims to promote the company’s transparency. The contemporary corporate governance structure requires a company to publicly disclose its information such as performance, ownership, sustainability, and financial health. Corporate governance also enhances company accountability and responsibility. For instance, corporate governance frameworks provide that board of directors should consist of independent directors representing shareholder and stakeholder interests. Shareholders shall elect all directors, and all directors owe fiduciary duties toward shareholders and corporations (Borgia, 2005; Saltaji, 2018). A study by Arulanandam (2020) highlighted that in empirical research, decision-makers in the corporate arena lack a true understanding of corporate governance and merely practice box-ticking in several cases.

### Neuro-Governance

Neuro-governance is a relatively new topic in social science. It is the result of the combination of neuroscience and governance. The primary function of neuro-governance is to reunify the fragmented social sciences such as political and economic science, social psychology, and the governance of social action subjects such as corporate governance, business administration, and public administration (Farmer, 2006, 2007). Before the emergence of neuro-governance, “should social science and social actions embrace neuroscience” was frequently explored by sociologists, psychologists, behaviorists, and other academicians. However, Farmer (2006) agrees that neuroscience significantly influences social sciences, social psychology, and social actions, which takes a further step than traditional methods.

The *neuro* in neuro-governance refers to neuroscience (Farmer, 2007). Neuroscience is a specialized discipline that studies the human brain’s relationship and central nervous system toward brain functioning activities such as decision-making, judging, thinking, behaving, and feeling. It is a discipline that consists of a gigantic field of neuroscience, for instance, behavioral and cognitive neuroscience, system neuroscience, cellular neuroscience, and molecular neuroscience (Farmer, 2008). However, the term *governance* here refers to the sense of governmentality. It is generally involved in a series of dynamically interrelated governing activities through a series of structured and systematic ways of rules, norms, and values (Farmer, 2007).

Nevertheless, neuroscience or social science alone is not sufficient to define and explain human nature and behaviors, human consciousness in perceiving, thinking, feeling, and judging, and decision-making processes (Farmer, 2007). The activities mentioned above do not solely result from neuron and dendrite interconnections but are also subject to human nature, philosophy, and psychological conditions. Therefore, neuro-governance could provide comprehensive views regarding the relationship among neuroscience, social science, and social psychology (Farmer, 2008).

### Why Neuro-Governance Is Important in Corporate Governance

There are at least two reasons indicated why corporate governance should embrace neuro-governance. First, neuro-governance could provide powerful supporting explanations for corporate governance behaviors and practices. Corporate governance is mainly involved in a series of complex decision-making processes. Neuro-governance could give comprehensive insights into how humans think, perceive, judge, and behave, effectively examining the minds of company directors and top management in decision-making and corporate governance behaviors (Farmer, 2008).

Second, human behaviors are not solely controlled by the brain and central nervous system neuro activities but are also substantially influenced by human nature, psychological conditions, philosophies uphold, culture, past experiences, and many other factors. It is a complex and sophisticated process. For instance, when humans face negative emotions, the amygdale in the brain will react to the immediate feelings and act below the level of consciousness until modified by the later signals from the cerebral cortex. Furthermore, human nature, such as greed and self-interest maximization, would also act as signals and markers to guide our behaviors (Farmer, 2008). Therefore, neuro-governance could provide comprehensive neuroscientific views that integrate all the factors mentioned above to explain human behaviors. Under the same circumstances, it could also examine corporate decision-makers’ underlying reasons and drivers for unethical behaviors and misconduct (Farmer, 2008).

#### Neuro-Accounting

Neuro-accounting is an emerging scientific way to study the relationship between accounting principles and functions of the human brain in making economic decisions and building economic institutions (Ahmad, 2010). Although there is not much accounting literature that presently links accounting principles and neuroscience, neuro-accounting suggests a strong parallel relationship between culturally evolved accounting and human brain activities in making economic decisions (Dickhaut et al., 2010). It argues that neurons coordinate human brain activities for gathering and evaluating information. The evolvement of accounting principles and institutions is no coincidence. The current longstanding accounting principles consistently emerge and persist in how the human brain reacts to economic decision-making (Dickhaut et al., 2009a, 2010).

#### How Is Accounting Related to Human Decision Making

Contemporary accounting perspectives originated from simple bookkeeping activities. Accounting’s primary function is to record the effects of economic and social exchanges. Of late, modern accounting has been evolving to provide systematic and orderly quantified estimations using monetary units for economic and social exchange opportunity costs (value received and sacrificed).

Accounting records provide past exchange memories to the human brain. These past exchange memories are the fundamental driver to sustaining human behavior norms of exchange. Human behaviors and decision-making would directly impact the past exchange memories of interaction, cooperation, performance, and experiences (Dickhaut et al., 2009a,b).

Specifically, memories display major behavioral norms that impact human reciprocity in decision-making, which are the social norms for fairness (Dickhaut et al., 2009b).

#### Social Norm of Fairness

The social norm of fairness generally assumed that humans would accept exchanges that could generate positive returns. It is based on the human nature of greed. However, Dickhaut et al. (2010) hold a contrary view and discovered that humans tend to reject exchanges that generate low benefits or are unfair in distributing returns. Their ultimatum game experiment distributed $10 as an economic pie to group 1. Group 1 has the choice to share their economic pie ranging from zero to the entire $10, with group 2, and group 2 can either accept or reject the share offered. As a result, they discovered that most group 2 respondents reject offers of $3 or less and tend to counteroffer a 50/50 split. This result rejected the assumption of the social norm of fairness. Unfair offers would activate human neuronal activities in the bilateral anterior insula, dorsolateral prefrontal cortex, and anterior cingulate cortex. The activation in the insula resulted in negative emotions (anger, dissatisfaction) and predicted responder rejections. On the other hand, the dorsolateral prefrontal cortex and anterior cingulate cortex activation are more related to less emotional, cognitive, and systematic processes to balance emotions and detect cognitive conflicts (Dickhaut et al., 2010; Hang et al., 2022).

### Neuroeconomics

Neuroeconomics is a relatively new neuro-governance discipline; it is the study of human economical behaviors and decision-making. It integrates the principles of neuroscience, microeconomics, and psychology to provide comprehensive examinations of human economic decision-making and behaviors (Ahmad, 2010). Traditional economic theories are typically concerned with optimal allocation of scarce resources. This assumption means that all economic decisions should reflect the principle of utility maximization by optimal allocation or consumption of scarce resources (Kalenscher, 2009; Sarfraz et al., 2021).

Zeki et al. (2004) further defined economics as the fundamental science of decision-making that could be applied to explain a wide range of corporate and human behaviors. The economic model of decision-making presumed that human evaluation of rewards relied on verbal reports and valuing possible actions to achieve the desired rewards (Brocas and Carrillo, 2008; Sarfraz et al., 2020). This economic model has been widely applied in public finance, law, and economics. However, neuroscience is an exquisite arsenal of scientific examination of human decision-making and behaviors (Abreu, 2010). Human decision-making is a complicated and sophisticated process involving a series of generic input-process-output-feedback activities resulting from a billion neuron interactions (Camerer et al., 2004). Furthermore, neuroscience further explains how emotions, thoughts, and feelings impact human brain activities of decision-making.

Zeki et al. (2004) discovered a parallel relationship between neurosciences and economics. Economics has provided and tested numerous behavioral models without further studying the underlying factors of behaviors, whereas neuroscience is a powerful institution for examining the black box of behaviors (Abreu, 2010). This could allow behaviorists and economists to answer fundamental questions about human behaviors, such as “why do two individuals make different decisions and behave differently although they have the same information?” and “why could the same person behave differently even though the circumstances are the same?” (Abreu, 2010).

Neuroeconomics is the natural extension of bioeconomics, which effectively integrates evolutionary biology and cognitive psychology to provide comprehensive examinations of ultimate factors that impact human decision making and behaviors and predict human behaviors (Abreu, 2010).

#### Neuroeconomics in Decision-Making

Risks and uncertainties are ubiquitous in human decision-making (Camerer et al., 2004). Most human decisions are associated with certain risks to achieve their desired outcomes and returns (Kalenscher, 2009). Risks and uncertainties primarily come from humans’ lack of opportunities and abilities to access and integrate all necessary information to predict future decision outcomes precisely and accurately (Platt and Huettel, 2008; Shah et al., 2021).

In addition, rapid changes in the environment and unexpected situations further deteriorate human abilities to make reliable and accurate conclusions or estimations for future decision outcomes. The impact of risks and uncertainties on the human decision-making process is subjective. It depends on the decision maker’s personality traits (risk-averse, risk-neural, risk-seeker) (Kalenscher and Pennartz, 2008). Risk-averse decision-makers generally dislike risks; therefore, they prefer lowest risk returns in exchange for lowest risks. On the other hand, risk-seeker decision-makers are generally intrigued by high-risk decisions, and they view high risks as an opportunity to realize high returns. Risk-neural is somewhere between risk-averse and risk-seeker. The risk-neutral person’s decisions are not subjected to risks and uncertainties thus. Their decisions are indifferent with equal expected payoffs (Kalenscher and Pennartz, 2008; Platt and Huettel, 2008).

Expected utilities are the common use indicator of how humans evaluate risks and uncertainties to make future options or decisions. It is a sum of a probability-weighted method to determine outcome distributions. This method assumes that humans inherently prefer options with the highest expected value (Platt and Huettel, 2008). For instance, A Berhad’s top management plans to undertake an investment between two options; option one has a probability of 0.85, and option 2 has a probability of 0.7. The expected utility theory suggests that top management would undertake option 1 with the higher expected value (Kalenscher, 2014).

However, the expected utility theory is insufficient to truly reflect real-world decision-making, even though it provides a powerful theoretical framework for describing how humans make decisions under uncertainty. It is because the distribution outcome in real-world decision-making is generally unknown. No one could predict decision consequences and results without knowing possible future outcomes, even probabilistically. Therefore, a decision is made under ambiguity and uncertainty (Platt and Huettel, 2008; Herrmann-Pillath, 2019).

Kalenscher (2009) discovered that most people are not risk-neutral when they make decisions under risks. They would systematically prefer lowest risk options (risk-averse) even though both options offer the same expected payoff. Furthermore, some people would systematically prefer risky options like gambling, because risky options could result in highest returns. Therefore, Kalenscher (2009) suggested that humans are inherently risk-seeker or risk-averse. This research finding is somehow consistent with the principle of personality traits toward risk attitudes, because different people have different risk attitudes (Kalenscher and Pennartz, 2008). From the uncertainty perspective, Kalenscher and Pennartz (2008) discovered that humans tend to be uncertainty-averse when making monetary decisions and uncertainty-seeking when faced with possible losses.

### Intertemporal Choice Theory

Intertemporal choice theory is concerned about decision outcomes that occur at different time points (Kalenscher, 2009). It holds a slightly different view from the expected utility theory, which suggests that preferences of immediate or temporally remote outcomes are not the sole function of outcome probabilities. Still, it also takes time to realize attitudes (Kalenscher and Pennartz, 2008). Desired outcomes or rewards delivered after a long delay are less attractive than the immediate ones. Therefore, intertemporal choice presumed that humans generally prefer short-term outcomes rather than outcomes that need a specific time to consume or realize.

Discounted utility theory is the mathematical formula commonly used in intertemporal choice theory (Kalenscher, 2009). This utility model is equivalent to the expected utility model in the domain of time attitudes (Kalenscher and Pennartz, 2008). It held a contrasting view with an expected utility, which assumed that decision-makers would base on the weight of temporal discount factors to choose options. However, expected theory presumed that the weight of outcome probabilities is the factor that influences decision makers’ choices. In addition, discounted utility assumed a constant discount rate for the discount functions. It translated similar EU axioms into the domain of time (Kalenscher and Pennartz, 2008). Stationarity is critical in which the decision makers’ preferences should be consistent over time. The preferences over two alternatives should be the only disparity between the time taken to realize the options. Therefore, it suggests that humans generally prefer options that have shorter realization time points (Pammi and Miyapuram, 2012). For instance, there are two options: option 1 is to receive RM 100 in 5 days, and option 2 is to receive RM 200 in 20 days; both intertemporal choice and discount utility suggest that humans would systematically prefer option 1 (Kalenscher, 2009).

### Human Brain Under Uncertainty

There is a direct relationship between probabilities and utilities toward the human brain. In a functional magnetic resonance imaging (fMRI) experiment, the respondents made economic decisions under different risks and uncertainties. The probabilities of correct decisions that could generate favorable rewards ranged from 60 to 100%. Platt and Huettel (2008) discovered that the respondent’s dorsomedial prefrontal cortex has a significant activation and is negatively correlated with reward probabilities. The activation was the distinct effect associated with evaluating potential outcome probabilities.

Furthermore, human probabilistic classification and evaluation tasks in which decisions should be undertaken were primarily based on the relative accumulation of information toward one choice. Platt and Huettel (2008) stated that increased option risks and uncertainties resulted in respondents’ insular, lateral prefrontal, and parietal cortices’ high activations. The activations reached maximal when there was equal evidence for each of the two choices. These activations in overlapping brain regions implicated that explicit information on probabilities is crucial for neural control systems to control behaviors and execute processing. Moreover, the posterior parietal cortex is vital for humans to judge probabilities, values, and rewards; it is the primary region contributing to calculations and estimations. Activation of neurons in the midbrain’s ventral tegmental, ventral striatum, and ventromedial prefrontal cortices is evoked by receiving a reward stimulus (expected utilities or potential outcomes). Then, dopamine neurons’ computation modeling response properties would generate a hypothesis to track outcome prediction errors that reflect expectation deviations.

Platt and Huettel (2008) also found that the respondents’ brain firing rate would temporarily increase to respond to unpredicted rewards and cues for future rewards. Nevertheless, the firing rate would remain constant to fully predict and decrease transiently when expected rewards fail to occur. A study by Zwart and Mündlein (2013) illustrated that neuroscience plays a vital role in business from a poststructuralist perspective. The study revealed that neuroscience improves performance, encourages innovation, influences human factors, and brings “science” into management.

#### Human Brain Under Risks

In an experiment on human decisions under risk, Platt and Huettel (2008) mentioned that the respondents’ insular cortex increased in activation when they made decisions over high-risk outcomes. Specifically, it is a “double-or-nothing experiment.” The respondents would choose between safer options to secure their current winnings or risky options to double their winnings at the risk of losing everything. Platt and Huettel (2008) found that when subjects chose the risky options, their right anterior insula increased in activations. Subjects who scored greatest in psychometric measures of neuroticism and harm avoidance even reached maximal activation in the insula.

Brocas (2012) further suggested that human avoidance of risks may be maladaptive under certain circumstances. For instance, in an investment option between safety bonds and volatile securities, the subject’s insular activations are high when they select the safer option (bond) over the high-return securities and remain stable when decision-making impairments lead to increased risk. Furthermore, insular activation could also effectively reflect the region’s supposed role in representing somatic states primarily used to predict an option’s potential negative consequences (Camerer et al., 2004).

### Anomalies in Choice Behavior

#### Reflection Effect

Expected utility suggested that human risk attitudes should be consistent with potential gains or losses. In general, an individual who preferred a gamble yielding RM10,000 with certainty to a risky gamble yielding RM20,000 with a probability of 0.75 would also prefer a same game-yielding loss with the same probability (Kalenscher, 2009). However, humans tend to prefer risky options during gambling to avoid losses. The reversal of risk attitude and choice behavior has been caused by the reflection of changing signs from gains to losses. This effect reveals that human choices of decisions are not made concerning final states but are significantly impacted by change with respect to gains or losses in the final stage. The subjective reference point would separate the domain of gains from that of losses.

#### Loss Aversion

Human cognitive processes in prospective gain are different from prospective loss when we choose not to play a mixed gamble. In a 50–50 gamble game, humans tend to play when gain magnitudes are more significant than two times than loss magnitudes. Thus, humans are more sensitive to the probability of losing money than winning money.

#### Non-stationarity in Time Preference

Several economists disagree with the stationarity assumption in intertemporal choice. In stationarity assumption, humans prefer receiving RM100 today rather than RM200 after a year. However, the economists argued that if the option is to receive RM100 today and receive RM200 in 2 months, would decision-makers still prefer the RM100? Reversal of human preferences could constantly occur when rewards and losses are advanced or shortened. The literal discontinuity of preferences could be repeatedly observed when immediate outcomes become available (Kalenscher, 2009).

### Neuro-Ethics

Based on the definition provided by Adina (2002), neuro-ethics can be defined in two content areas: ethics for neuroscience and neuroscience of ethics. This research would be solely focused on the latter. Neuroscience of ethics would provide comprehensive examinations of how traditional themes of ethical values and moral philosophies could impact human activities. On the other hand, ethics for neuroscience mainly concerns ethical issues in neuroscience, such as potential impacts of research results on existing social, cultural, ethical, and legal structures.

The International Neuroethics Society further defines neuro-ethics as a contemporary field that studies human behaviors and decision-making processes and enhances human self-understanding by integrating the principles of neuroscience and related sciences of the mind (ethical and moral philosophies and human psychologies) (Beauregard, 2018).

#### Theoretical Themes for Neuro-Ethics

The following will cover four fundamental neuro-ethics themes to examine the differences between ethical decision-making and other types of cognition, the unconscious dimension of ethical decision making, the role of emotion and intuition, and neural-based normative ethical theories.

#### Not Just Another Decision-Making Process

Human neurocognitive decision-making and judgment processes are distributed toward different systems involved (different regions of the brain). These different regions would eventually integrate and act jointly when humans perform specific functions (Salvador and Folger, 2009). Therefore, several researchers and psychologists generally presume that human ethical decision-making is similar to other decision-making processes.

However, neuro-ethics holds a different view, which suggests that humans’ ethical decision-making is distinctive from other forms of cognitive and decision-making processes. The medical case of Phineas Gage has evidenced this view. After Gage suffered a brain injury, he maintained motor control and sensory perception. On the other hand, his social behaviors significantly changed, making him an alcoholic and unreliable at work. Over time, he even lacked moral sensibility, prudence, and self-control. Therefore, this medical case evidences that humans’ ethical decision-making and moral judgment are dissociable from the other form of thinking and decision-making processes in which the ethical decision-making appeared as an independent intellectual ability, and its neuroscience activities can also be distinguished from other mental processes (Salvador and Folger, 2009).

In a neuroimaging experiment, Mendez (2006) stated that certain areas of the human brain have high levels of systematic activation when subjects engage in moral judgments, cognitions, and appraisals. For instance, there will be high levels of activation in the frontal lobe (medial orbitofrontal complex, dorsolateral prefrontal cortex) and subcortical-limbic structures (amygdala, hippocampus). This experiment showed that certain areas of the brain would have high levels of activation when the decision-maker responded to ethical-related stimuli, social perception, and norm compliance. Furthermore, Salvador and Folger (2009) found that specific brain regions of participants have a high degree of activation when they formulate behavioral intentions and moral judgments toward ethical and relevant decisions.

Finally, there are consistent results from numerous neuro-ethics studies from the ethical behavior perspective. For example, the amygdala and ventromedial prefrontal cortex have a greater degree of activation when decision-makers act in ethical manners.

#### More Than Just Conscious Reasoning

Neuro-ethics argues that human ethical decision-making involves more than just conscious reasoning (Salvador and Folger, 2009). This assumption is consistent with the dual processes of human social cognition models (controlled and automatic). “Controlled” is the conscious and intentional cognitive process, whereas “automatic” is non-conscious and spontaneous. Based on the social and cognitive psychology contexts, humans would automatically categorize and interpret what they sense in their immediate environment. These categorization and interpretation processes include physical objects and the meaning of social behaviors and often occur without human awareness (Bargh and Ferguson, 2000). In other words, these processes are beyond intentional human control.

Furthermore, these processes are mainly dominated by human past experiences, emotions, norms, and moral values (Reynolds, 2006). These variables unconsciously constitute implicit associations between concepts and eventually become human assumptions for decision-making. Therefore, these automatic processes arguably dominate a large part of human life compared to controlled processes. Based on the dual processes model, Lieberman et al. (2002) introduces a reflective-reflexive model to examine how the dual processes model could impact human ethical decision-making. In this model, ethical decision-making is separated into two primary cycles: the reflexive pattern matching cycle and the reflective process. The reflexive cycle is primarily influenced by stimuli (elements of everyday experiences, emotions, norms, and moral values) sensed by brain activation processes. Specifically, when humans face potential dimension situations, the pattern matching cycle will occur automatically without consciousness. The human brain would organize and structure these stimuli into neural forms and then compare them against ethical situation prototypes. Other parts of the reflexive support system (X-system) would undertake these iterative and cyclic stimuli processing activities and seek more stimuli until the situations match the decision maker’s general prototypes for the ethical issue or scenario.

Hence, the human brain will undergo a process to present the current situation and provide normative evaluations and prescriptive recommendations for decision-makers to act reflexively according to the ethical situation.

Sonenshein (2007) proposed the sensemaking-intuition model of ethical decision-making based on elements of the dual processes model. Under this model, there are three primary phases in ethical decision-making: issue construction, intuitive judgment, and explanation and justification. Sonenshein (2007) further explained that the ethical decision-making process begins when decision-makers attempt to interpret the ethical situations they face and engage in ethical issue construction. After plausible interpretation of situations, the decision-makers would make instantaneous and effortless intuitive judgments based on their respective moral intuitions. After that, they will explain and justify their judgments or interpretations by conscious moral reasoning toward themselves and others (Sonenshein, 2007).

Furthermore, various empirical studies show that moral intuitions and subconscious elements of moral judgments directly impact ethical behaviors. Reynolds et al. (2010) found that businesses behave intrinsically ethically (such as safeguarding shareholder’s interests at the expense of stakeholders) which promotes company decision-makers to make unethical decisions. Reynolds et al. (2010) measured the respondents’ intensity toward inherent morality of business implicit assumption with business-associated terms (such as CEOs, board of directors, profit maximization, and resource optimization) and ethical behavioral terms (such as equality, fairness, responsibility, and following of rules) and subsequently measured how fast the respondents could associate the terms with unethical behaviors (such as lying, stealing, fraud, and misappropriation). As a result, Reynolds found that respondents with strong business ethics and implicit assumptions were more likely to engage in unethical behaviors (financial statement manipulation, tax evasion, and misuse of company assets) at the unconscious level. Therefore, this study at least proves that unconscious and implicit judgment could explain the underlying reasons for unethical behaviors under certain conditions.

### The Emotional Component

Hauser (2006) summarized his neuro-ethics research with a short sentence *”If it ain’t got emotion, it ain’t got moral swing.”* Damasio (1994) proposed the concept of the “Somatic Marker Hypothesis.” This hypothesis model suggested that the unpleasant and gut feeling form of the human internal “alarm” mechanism could prevent us from adverse outcomes even before undertaking any rational analysis. In other words, human ethical decision-making is not merely influenced by intuitions but also mainly contributed by emotional components (Berthoz et al., 2006).

The primary human brain areas involved in affective and emotional processing are the medial orbitofrontal cortex, anterior cingulate, and amygdala from the neuroscience perspective. when decision-makers encounter moral- and non-moral related stimuli (Aamodt and Wang, 2009). Moll et al. (2001) randomly distributed picture sets toward subjects in an fMRI study on neural reaction toward moral awareness. The sets included pictures of moral violations (physical maltreatment of animals) and non-moral relevant “neutral” pictures of landscapes and machinery. Moll et al. (2001) measured the subjects’ emotional responses and neural activations. Subsequently, it was found that the subjects’ emotions had been evoked and the brain regions mentioned above had significantly high activations when they observed the morally relevant pictures. These results showed that emotions had played critical roles in part of human moral recognition dimensions under certain circumstances.

Various studies demonstrate that emotion plays a critical role in moral awareness and moral judgment formulation. For instance, numerous behavioral and neuroimaging experiments suggested that specific brain regions increased in activations when participants were asked to make moral-related stimuli or moral dilemma judgments (Salvador and Folger, 2009). Therefore, suffering injuries, tumors, and lesions are consistently impairing moral behaviors and judgments (Eslinger and Damasio, 1985). Like the Phineas Gage case, damage in the ventromedial prefrontal region has diminished his ethical decision-making capabilities.

Furthermore, some empirical neuro-ethics studies suggested that human moral judgment neural systems are correlated with certain emotions (fairness, judgment of fairness). Tabibnia and Lieberman (2007) suggested that human moral judgments will be unconsciously impacted by deep-seated or emotions. Incorporating emotional components into human ethical decision-making seems critical. The absence of an “appropriate” emotion could provide supplementary examinations of unethical behaviors among people who engage well in “normal” moral reasoning. Reynolds (2006) suggested that brain systems for ethical behavior abstract rule storage are different from those for rules assessment and application.

Despite emotions playing a critical role in ethical decision-making, the extent to which emotional processing depends on moral or ethical dilemmas that decision-makers face. Greene et al. (2001) subsequently found that when respondents engage in moral judgments, their brain regions associated with emotions have higher activations than regions related to their working memory. Nevertheless, it was merely true among respondents that were assigned to consider what they labeled as personal and impersonal moral dilemmas. Greene and Haidt (2002) suggested that emotions will be only involved in personal moral judgment (such as potential to cause bodily harm to other people in the way that such harm does not result from different party redirection of impending threats).

In a brief conclusion, emotional processing plays a vital role in ethical decision-making (Salvador and Folger, 2009). Some neuro-ethic studies evidenced moral judgment involved certain emotions and conscious reasoning non-linear interactions (Reynolds, 2006). In the same concept, when company decision-makers perceive conflicts between competing duties and obligations, their brain regions associated with emotional and moral reasoning seem to be activated and automatically participate in the process (Hauser, 2006).

### GAGE Model for Conflicts of Interest Examination

Thagard (2007) introduced the GAGE computational model to examine the integration process between cognitive information and emotional information. This model comprehensively integrated cognitive activity regions like the hippocampus and prefrontal cortex and somatic-driven activity regions such as the amygdala and nucleus accumbens to examine human decision-making processes. It concluded that emotions result from the ventromedial prefrontal cortex’s dominant and interconnected activities. These different regions would integrate judgments of situations, goals, and predicted rewards with current physiological states of non-verbal representations. Thus, emotions are both cognitive and physiological.

Furthermore, this model argues that moral judgment is neither purely cognitive in ways the traditional utilitarian theory proposed nor purely emotional in ways expressivists and emotivists have supposed. Still, all human thinking and reasoning activities, including moral reasoning and decision-making, are cognitive- and emotional-based. To apply this model to the conflict of interest perspective, Thagard (2007) further provided the case of RIM Park. In 1999, Waterloo City Council decided to build a sports facility. The city council adopted a finance lease of $112 million from MFP, which will be paid back over 30 years. After a few months, the actual finance lease cost was revealed as $227 million, and then the city council took legal action against MFP for scamming the city. Investigators subsequently found that the city treasurer, John Ford, had not thoroughly read the lease contract’s final version. The city’s chief administrative officer, Thomas Stockie, had violated the conflict of interest policy. Stockie had attended numerous social events with MFP’s vice president, David Robson and alleged that the attendances were to build good relationships with partners. He did not get any interest from MFP. Stockie further alleged that he did not thoroughly check prior approval because he trusted Robson. As a result, Ford and Stockie were guilty of conflict of interest, and the poor judgments in this deal were partly due to the friendship with Robson and extensive socializing paid by MFP.

From the GAGE model implication, Waterloo executive’s bad decisions arise from the unconscious cognitive or effective process. They seemed to become associated with positive somatic markers, with Robson and his deal mainly caused by pleasant social and professional contacts. For instance, because of positive anticipations of pleasurable situations with Robson or probably fear of disappointing their buddy, Ford and Stockie breached their official duties to examine and execute the contract prudence, and a conflict of interest arose.

Based on the RIM case, Thagard (2007) proposed that decision-makers with acquired interests in their official duties seem to be unreliable or incapable of determining whether their decisions emanate from biases they have acquired from personal interests than reasonable and systematic reasoning from their official responsibilities. They would also fail to decide whether or not they act appropriately or out of a conflict of interest. Therefore, Thagard (2007) suggested that humans would act immoral or unethical because of conflict of interest under certain circumstances.

#### Role of Financial Incentives

Moore et al. (2010) studied how financial incentives could contribute to conflict of interest and intrusion of bias. In this experiment, respondents were assigned different roles (buyers, buyer’s agents, sellers, and seller’s agents) and were given the same information about the sample company (E-Settle). The agents have to review the information and offer an unqualified endorsement from the principal’s assessment or they need to make their assessments. Subsequently, the principals would review the agents’ reports and negotiate for the purchasing of E-Settle based on their own opinions and the agents’ estimations. As the agreed-upon price went up, sellers earned most of the profits, and buyers earned the least (Moore et al., 2010). At the same time, all the agents were required to report their own beliefs in the target company value, and their valuations shall be as just and as impartial as they can. Their revaluation values will then be compared with the non-partisan experts’ opinions. The experts estimated the target company to be worth $14 million, and if their estimations were within $3 million of the experts’, they could receive an additional $3. After that, the respondents were asked to express their confidence in the appraisal’s accuracy. They were also allowed to bet their appraisals; if they chose to accept the bet, they could win more money ($6 instead of $3), but their appraisals needed to be more accurate (within $1.5 million instead of $3 million) (Moore et al., 2010).

Furthermore, Moore et al. (2010) also found that the conditions mentioned above would continuously impact the people, although they had already stepped down from the agent role. They may be aware of their biases in decision-making but tend to underestimate them. In the experiment above, almost all the agents believed that their roles comprehensively influenced their assessment as agents, but they also underestimated the power of biases and financial incentives (Moore et al., 2010).

#### How Self-Interest Results in Unethical Behaviors

Contemporary ethical theories describe unethical behaviors as immoral or illegal actions that would eventually cause great negative impacts on humans. Grover and Hui (1994) suggested that the human nature of self-interest is the fundamental key to unethical behaviors (stealing, cheating, and lying). Humans usually tend to lie to obtain their desired interests and benefits. It is consistent with the agency theory researched by Trevino (1986); it was found that agents would typically lie to their principles when they are in advantageous positions in corporate internal information assessments to obtain desired interests and benefits.

Furthermore, Grover and Hui (1994) integrated both self-interest and role conflict interest to examine unethical behaviors. Specifically, role conflict of interest is more likely to lead to unethical behaviors when a person could obtain benefits from particular behaviors. Self-benefit maximization will further lead to greater unethical behaviors. Expected rewards invisibly increase the effect of role conflict, because it is generally difficult for humans to satisfy two confronted demands simultaneously. Although some people may consider various options to resolve a conflict, humans tend to fulfill one role demand under normal circumstances and ignore another role of demand. Therefore, humans may be motivated to conduct unethical behaviors when their desired rewards are attached to unethical behaviors. They are more likely to conduct these unethical behaviors when an acute role conflict prompts.

Grover and Hui (1994) proposed that the desired reward is critical for humans to evaluate alternatives (resolved by choice, compromise, or avoidance). When a reward that could be gained from unethical behaviors is great, it is more likely that humans would respond to role conflict and conduct such behaviors. On the other hand, if a person is faced with role conflicts without any desired interest, he/she will be more likely to resolve the conflict in another fashion. Therefore, rewards are a negative reinforcement that could motivate humans to conduct unethical behaviors.

Nevertheless, Etzioni (1988) disagreed and criticized the ground of self-interest theory. He argued that the human norms of behavior are beyond external reinforcement contingencies. He provided different views on circumstances in which people behave ethically and are not easily influenced by desired rewards and interests. For instance, some people have an ample opportunity to obtain desired interests and benefits using advantageous positions undetectably. Still, they choose not to do it because of personal values and beliefs. Rasool et al. (2021) argued that wrong decisions might have roots in a toxic working environment, which permeates negative vibes in an office. The well-being of employees has a significant influence on their behavior patterns, coupled with poor engagement with the organization. Rasool et al. (2020) discovered that toxic culture would jeopardize work performance, which influences poor or biased decision-making. Rasool et al. (2019) studied bank employees in China, and long-time serving staff are more comfortable using old working methods, hence lacking innovative capabilities. However, such old methods were often trusted with high ethical values.

## Methodology

This research adopted the descriptive qualitative research approach where the researcher solely collected and interpreted the non-numerical responses from the targeted respondents to address the research objectives. The researchers selected a qualitative instead of quantitative approach as the research method, because statistical data or correlative relationships alone are not sufficient to provide a reasonable understanding of the phenomenon of contemporary corporate governance from the view of neuro-governance. Moreover, since it is an exploratory study, a qualitative study would be able to dive deep into its objectives and provide fruitful findings.

In the earlier stage of this research, the researchers accessed numerous related field journals to determine the research direction, obtain independent variables, and survey questionnaire drafting. The survey questionnaires were open-ended, primarily focused on “what and why,” so the respondents were required to express opinions and reasons. After that, the researchers conducted one-to-one structure interviews for the targeted sample to fill up the questionnaires from different selected areas.

### Sampling Design and Selection

The researchers adapted a non-probability sampling design in which they cannot guarantee that all segments within the population can be represented in the designed sample. Most of the population will have no opportunity to be sampled. In particular, the researchers adopted a purposive sampling technique. They specifically selected people at the managerial level (assistant manager, manager, general manager, and director), as the topic for this research is concerned with corporate governance and business practices. Furthermore, the targeted areas to conduct the survey were business offices and co-working spaces within Kuala Lumpur city, specifically Bangsar South, Bukit Bintang, and Bandar Utama, Kuala Lumpur, Malaysia.

### Conduct of Interviews

There were 21 structured interviews conducted between February and March 2021. Initially, all the interviews were supposed to be face-to-face in the respondents’ offices. However, only ten face-to-face interviews were conducted because of the coronavrius disease 2019 (COVID-19) pandemic and Movement Control Order (MCO) 2020; the remaining was conducted online via WhatsApp and normal phone calls. Nevertheless, the events above did not impact the sample selection areas. The researchers went to Bangsar South, Bukit Bintang, and Bandar Utama business offices and co-working spaces to obtain interview permissions before the MCO. Therefore, the samples were valid from the areas mentioned above. While the sample remains small, this study is exploratory, and the use of a qualitative approach is suitable for this study.

The number of interviews conducted seems small, but it has been sufficient for this research as the researcher’s concern was on quality rather than quantity. The respondents that took part in this research were entirely individuals. Most of the interviews were conducted between 50 and 90 min.

The respondents were given the greatest degree of flexibility to answer the questions. They were encouraged to express their ideas and opinions during the interviews. However, when the respondents were not comfortable answering the questions or showed unfamiliarity with any of the questions, the researchers would brief them on some basic ideas or theories to enhance their understanding of particular questions.

## Findings and Discussion

### Neuro-Governance Perspectives

The respondents were required to describe their understanding of neuro-governance by describing whether they had heard the term “neuro-governance” before and describe what neuro-governance is if they had heard of such a term. After that, they were required to express their opinions on how neuro-governance could provide detailed explanations and predictions on corporate directors’ behaviors and mitigate the corporate directors’ unethical behaviors.

As a result, it is surprising that there is only one respondent who has heard about neuro-governance from his psychologist friend. The remaining respondents indicated that they had never heard of such a term before the interview. When the researchers described that neuro-governance is a new discipline that combines various perspectives to study human behaviors and decision making, the first response from the respondents were, “is neuro-governance similar to psychology?” This common response indicated that psychology is still in the mainstream to study the decision-making process.

However, the only respondent that has heard neuro-governance before provided the following description:

*“In my understanding, neuro-governance consists of various theories to explain human behaviors. In view of psychology, only human behaviors are studied based on the psychological viewpoint. However, they tend to exclude areas such as economics, moral values, and accounting.”*

This respondent pointed out the limitations of the psychology discipline. For instance, psychology theories are traditionally concerned about how internal factors such as personality and emotion impact human decision-making, but these theories commonly may tend to undermine the impact of external factors like economic, accounting, and financial incentives. On the other hand, neuro-governance has comprehensively integrated both essential and significant internal and external factors to study human decision-making and behavior. Therefore, neuro-governance is more relevant and reliable now.

Other respondents also gave similar explanations when they addressed whether the neuro-governance theory is essential to explain and predict corporate director behaviors and practices. One of the respondents explained that:

*“Yes, I think neuro-governance is essential to explain and predict human behavior because neuro-governance is concerned with how humans perceive and make decisions under different circumstances. There are no uniform criteria for humans to make decisions. Human decisions will keep changing in different circumstances, combining all essential areas to provide a deeper understanding of the human decision-making process.”*

Human decision-making is a complicated process subject to various reasons like personalities, personal values, emotions, moral values, and financial incentives. Every human could make different judgments and behave differently under the same circumstances. Therefore, solely studying human behavior from a single perspective may be too biased and subjective. For example, one of the respondents described that:

*“Study on human behaviors based on a single viewpoint is too subjective. Every person could have different judgments, and neuro-governance is more reliable and objective to study and discover human behaviors.”*

Lastly, all the respondents agreed that corporate governance unethical behaviors and corporate misconduct could be mitigated by understanding how humans think, perceive, judge, and behave. Those in senior positions mainly commit corporate misconducts and unethical behaviors, and their behaviors are always impacted by their thinking and judgment. By truly and deeply understanding how humans think, perceive, and behave, common drivers or circumstances that could motivate corporate decision-makers to breach their fiduciary duties and misconducts can be detected. For example, one of the respondents explained that:

*“Every human action comes with meaning and purpose; if we have ways to understand how a human mind behaves, it is possible to mitigate unethical behaviors.”*

### Neuro-Accounting

Generally, all the respondents believed that past accounting records could enhance corporate transparency by providing a source of explanation about the business judgments. Past accounting records or financial statements enhance the transparency in which the company shall disclose all necessary information. Through these financial statements, the principals, shareholders, and stakeholders can assess the company’s health. For example, one of the respondents claimed that

*“By looking into accounting records, outsiders can know that the management decisions or business judgments whether are good or otherwise.”*

Therefore, the company should uphold high integrity and publish proper disclosures to gain market confidence. However, the function of past accounting records does not stop there. Past accounting records represent the experiences or lessons that the company itself learned before. Precisely, whatever mistakes the company made will be mirrored in these records. Giving weight to the above remarks is the following explanation from a respondent:

*“Yes. The past accounting records enable the stakeholders to make the right decisions. So, the stakeholders and shareholders will get to know any transactions with any suppliers which bring benefits to them and avoid any irresponsible business deal that occurred before.”*

During the interviews, the researchers discovered that the social norm of fairness is a quite controversial assumption in which there are different responses from different respondents. Specifically, 66% of the respondents agreed that humans would accept the exchange that could generate positive returns, 14% partially agreed, and 20% disagreed with the assumption. Respondents who agreed with this assumption support the behavior that humans are inherently selfish and greedy and would not reject any exchange that could benefit them even though such an exchange is illegal or unethical. Some of the respondents have put forward the following direct illustrations to support their opinions:

*“Agreed. Because this is part of human nature, selfishness”.*

*“I agree. This is part of our nature. We will do whatever we can to get, what we want as long as we can afford what it takes.”*

*“Yes. I totally agree with the concept of the social norm of fairness. Humans would not reject the exchange that generates positive returns even though it is a minor return.”*

Therefore, the social norm of fairness somehow showed that the human nature of greed and selfishness would motivate corporate decision-makers to engage in any exchange that could benefit themselves, although it is unethical and illegal. Furthermore, it also explained the underlying reason that could motivate corporate decision-makers to breach their fiduciary duties and pursue personal interests at the cost of the company, principals, and shareholders.

However, respondents who disagreed with the social fairness assumption stated that some things are beyond benefits and interests, for instance, moral values, personal beliefs, and religious teachings. Therefore, they will not feel motivated to conduct some things that are contrary to their values and beliefs, although they can obtain favorable returns from the actions. As one respondent stated:

*“No, because some things are more important than benefits or positive returns. For example, owned beliefs and religious teachings will guide us on what we can and cannot do. I will not do anything wrong in my belief.”*

For respondents that partially agreed, they stated that exchanges or transactions must be legitimate. In other words, exchanges or transactions shall be naturally legal under existing laws and regulations. If the exchanges are naturally legal, they will feel happy to engage in such exchanges, although the positive returns are minor. However, if the exchanges are illegal, they will stay away from these exchanges, because these transactions may be subject to serious penalties in the future.

On the other hand, all the respondents agreed that accounting principles could impact corporate decision-makers’ business judgments. For instance, humans dislike suffering losses in exchanges or transactions, and the expense matching principle provides how much we must sacrifice to obtain desired returns. If the returns received are greater than what we sacrificed, we will feel motivated to engage in the exchanges. However, we normally reject transactions, because the sacrifice is greater than the return. Such a principle provided a significant comparison basis for decision-makers to evaluate business judgments. One respondent claimed the following statement:

*“Expense is the cost to spend to earn the profit when the cost is more than revenue, meaning is lost. Humans are not like engaging in transaction debt that causes fussed loss.”*

Furthermore, some of the respondents even suggested that the revenue realization and expense matching principles enhance the comparison and improvement ability. The company’s financial performances are outcomes of these two principles, and the corporate decision-makers easily compare the performances with other companies within the same industry. When competitors report a good performance, there must be critical success factors that make them successful, so corporate decision-makers can learn from them for future improvements. Therefore, business judgments could be enhanced in the future. For example, one of the respondents explained that:

*“Yes, because when the company is suffering a significant loss, there is something wrong in the decision making. So that the accounting principles reflect past information and provide an opportunity for future improvement.”*

In a brief conclusion, all the respondents agreed that neuro-accounting had enhanced the corporate decision makers’ business judgment and corporate governance quality. First, past accounting records provide historical and current information for users to make economic decisions. Second, accounting principles are essential to provide fundamental analysis criteria for decision-makers to make business judgments. Third, past accounting records and accounting principles offer reliable and relevant alternatives for future improvements. From the corporate governance context, past accounting records at least draw some evidence that external parties could rely on to evaluate company performances and management judgments and facilitate check and balance alternatives to prevent corporate misconducts.

### Neuro-Economics

Economic theories are mainly concerned with how people optimize the allocation of scarce resources to achieve maximum utilities. This is the fundamental principle in the economy. However, 90% of the respondents believed this basic economic principle is one reason that motivates corporate decision-makers to conduct unethical behaviors.

Specifically, this is a common problem in the agent-principal relationship. When principals appoint managers to direct a company on their behalf, they already expect that the managers will contribute maximum wealth and returns to them. The problem here is that the management is generally facing economic resource constraints, and such constraints may limit their abilities to achieve goals and objectives. When the management fails to satisfy the principal’s requirements, the principals may have the power to reduce their remunerations or even remove them from management positions. Therefore, to secure their well-paid remunerations and positions, they may be encouraged to use their professional knowledge to deceive the principals and shareholders. For example, they may recognize revenues in advance, manipulate asset useful life, and defer recognition of expenses and liabilities.

One of the respondents remarked the following statement:

*“Yes. If the management faces a very strong resource constraint, they face a lot of pressures in which they need to use the limited resources to create maximum wealth for the company and its shareholders. When they fail to achieve it, they may conduct unethical behaviors to cheat the shareholders.”*

On the other hand, the remaining 10% of the respondents rejected the assumption. They argued that the management had owned fiduciary duties toward principals in the agent-principal relationship. Therefore, they shall exercise their fiduciary duties objectively and professionally. For instance, the management should use their professional judgments to ensure that all available scarce resources are consumed effectively and efficiently. There is no reason to legalize unethical behaviors.

In a similar vein, there is one respondent who remarked that:

*“No, since the company faces limited resources, the management shall utilize and consume these resources effectively and efficiently. So, it is not an excuse for them to conduct unethical behaviors.”*

From the level of risk, uncertainty, and expected reward perspectives, all the respondents agreed that these perspectives would influence the corporate decision makers’ business judgment rationality. Human personalities toward risks and uncertainties came into the picture. Risk-takers would prefer high risks to generate high expected returns. The risk-averse would naturally prefer safer and stable options to protect themselves, although the expected returns are low. However, human personalities may be distorted in the agent-principal relationship. Managers are always facing profit maximization pressures from principals and shareholders. Under these circumstances, they may feel motivated to engage in high-risk options to generate maximum returns, as any loss will be eventually borne by principals and shareholders, not them. Therefore, to a certain extent, the risk, uncertainty, and expected reward perspectives explain why managers undertake high-risk options at the cost of principals and shareholders.

As one of the respondents remarked:

*“Yes. If the expected reward involves greater risks and uncertainties, there need to be greater rewards to compensate for such risks and uncertainties. When the decision-maker faces pressure from the principals, the decision-makers may want to invest in high risks for greater returns.”*

Expected and discounted utilities are the most controversial principles in the neuro-economics section, and there is a huge gap in the respondents’ judgments. Specifically, 43% of the respondents supported that expected utilities will commonly affect corporate decision-makers’ business judgments. Expected utility is a sum of the probability-weighted method to determine the distribution of outcomes. High outcome probability means low risks and uncertainties. Therefore, expected utilities could provide a greater picture of risk comparison for decision-makers to evaluate each option. One of the respondents explained that:

*“I think expected utilities will commonly impact decisions because the probability is important for the company to make decisions. The company should undertake a high probability option, which a high probability means low risks and uncertainties.”*

Therefore, Platt and Huettel (2008) suggested that humans generally prefer options with highest expected value. However, it is surprising that 48% of the respondents prefer discounted utilities. In their views, immediate or temporally remote outcomes are not the sole function of outcome probabilities but the time taken to realize attitudes. Nevertheless, it was very interesting that although they agreed with the fundamental discount utility theory, at the same time, they also rejected the discount utility assumption that humans generally prefer short-term outcomes rather than outcomes that need a certain time to consume or realize. In their opinions, they agreed with the assumption above only when both options have the same probability outcome and expected rewards, just different in the time frame to realize. However, there will be a different story when both options offer different expected rewards due to different times to realize. For example, receive RM100 in 5 days or RM500 in 20 days. People who demand greater returns would automatically prefer to receive greater returns later, although a long time to realize may result in greater risks and uncertainties.

Concerning the discussion above, the respondents made the following comments:

*“Yes, my goal is to maximize the return. I don’t mind taking risks at a longer time to receive more returns.”*

*“Yes. For example, I am a risk-averse person. Still, when I have two options now, one is to receive RM1000, and another one is to receive RM2000 next week, I will prefer the second option because a short timeframe can result in a huge difference in return despite a longer time to realize may result in greater risks and uncertainties.”*

Therefore, they stated that discounted utilities make more sense in business when the company faces limited resources to maximize utilities. Besides that, discounted utilities, to some extent, explain why unethical managers are willing to involve more risks and uncertainties to maximize their performances. The remaining 9% of the respondents did not support expected or discounted utilities. They were more concerned about human risk attitudes, levels, and expected returns.

Finally, 90% of the respondents agreed that anomalies in economic choice behavior would lead corporate decision-makers to make poor and unreliable business decisions. There are three anomalies discussed during the interviews. First, the respondents agreed that reflect effects are the main anomalies that lead decision-makers to make unreliable decisions. In their views, humans would naturally prefer risky options when suffering losses. When we incur losses (either a minor or significant loss), what we think usually is not how to minimize our losses but to take the opportunity to cover the losses (some may even wishfully think to earn profits from the losses). One of the respondents gave the following classical illustration of the reflection effect:

*“We should quit the gamble games after losing all of our initial capitals to minimize the losses. However, we can see many people will put in more money or even borrow money in hope of covering the losses and earning something extra. It is extremely dangerous that such risky options result in great risks and losses.”*

The illustration above sufficiently explained how humans are naturally risk-seeking in the domain of losses. To a certain extent, this reflection effect illustrates the managers’ risky behaviors to maintain their performances in the eyes of the principals. Second, although the respondents do not understand much about loss aversion, one respondent highlighted that this anomaly positively affects decision-making. Loss aversion assumes humans are more sensitive to the probability of losing money than winning money. When we have such mindsets in our minds, we will not simply make decisions without proper judgments. Therefore, we will be motivated to do more preparation work and research before making decisions. Third, the respondents agreed that non-stationarity in time preference could distort human personality toward risks and uncertainties. For instance, the intertemporal choice assumed that the expected returns delivered after a long delay are less attractive than immediately. However, when another option offers a higher return in a slightly delayed time, the risk-averse person’s attitude may be distorted. They could obtain a far higher return by accepting a small increment of risks. For example, a risk-averse person may reject the option of receiving RM500 next month instead of receiving RM100 today. However, when the option has changed to receive RM500 in the next 3 days, that risk-averse person’s preference might differ.

On the other hand, respondents who declined the abnormalities assumptions rejected the reflection effect theory, which states that humans are inevitably risk-averse to a loss. There is a reason that we need to rely on expected utilities or discounted utilities to determine the level of risk before, making certain decisions. Therefore, they strongly believed that humans would not be motivated to engage in risky alternatives after they suffered losses. Instead, they will be taking profits when there is any gain and immediately cut losses when they suffer losses. The discussion above can be witnessed from the following remark:

*“No, because we have already suffered loss, it is very dangerous to engage with the unnecessary risks that would cause more losses.”*

### Neuro-Ethics

Generally, all the respondents agreed that corporate ethicalness would be primarily influenced by corporate decision-makers’ emotions, past experiences, and moral values. The respondents stated that negative emotions like fear and anger would significantly influence our logical and rational thinking from the emotional context. Such logical and rational thinking are the most important forces for people against any unethical temptation or desire. Negative emotions would lead people to make irrational decisions without proper judgments. Instead, we make decisions emotionally. Some of the respondents even argued that emotion overrides our moral values. One argued that moral value application requires a significant level of proper and rational judgments. When we lose the capability to analyze facts and circumstances objectively, how can we apply our moral values into consideration? For instance, one remarked that:

*“Yes. I agree corporate ethicalness would be influenced by corporate decision maker’s emotions, norms, and moral values. First, we will lose our rational thinking when facing negative emotions. For example, when we are angry, we might lose control in our behaviors. It is totally the same in the ethical situation. So, when we face negative emotions, I don’t think we will still follow the moral values.”*

Besides that, almost all the respondents stated that the corporate directors’ moral values are directly related to corporate ethicalness. Our moral values are the steelyard in our minds that provide parameters to guide us on what is right and wrong. In other words, moral values are powerful and significant perspectives that helps us distinguish between right and wrong and ethical and unethical. Therefore, human behaviors will be ultimately impacted by personal moral values. For instance, people who uphold strong moral values are unlikely to engage in unethical behaviors to pursue their desired interests. They will feel guilty about pursuing their interests at the cost of others.

In addition, some of the respondents suggested that humans would inevitably bring self-beliefs or values where they are. For instance, when the top management uphold high moral and ethical values, such values would be invisibly injected and integrated into the organizational culture. The attitudes and behaviors demonstrated by the top management will eventually become the role models for employees. On the other hand, if the top management demonstrates negative behaviors like laziness and dishonesty, the employees will also be influenced by their behaviors.

Giving weight to the above discussions is the following illustration from the respondents:

*“Agree. For example, if the decision-maker faces pressure from shareholders for a long time in the emotions part, he may lose control and do things irrationally due to negative emotions. Thus, he may make unreliable decisions like engage in unethical behaviors.”*

*Yes, employees only execute decisions made from their superior. Superior is the one who makes decisions. So, if superiors don’t have the so-called “sanity” to discipline themselves, how good can corporate ethicalness be”?*

Therefore, it is probable that top management that lacks moral and unethical values would be motivated by their interests to conduct unethical behaviors at the cost of principals and shareholders. As the saying goes, “fish begins to stink at the head.” If the management has displayed negative behaviors, how good can the employees and corporate ethicalness be exhibited?

On the other hand, all the respondents agreed that the corporate decision makers’ assumptions and moral intuitions would impact corporate ethicalness. Like moral values, personal assumptions and moral intuitions are underlying rationales that commonly impact human decision-making. Specifically, we act as what we believe, and our behaviors constantly reflect our beliefs. Positive personal assumptions and moral intuitions would generally motivate people to conduct themselves positively. In contrast, negative personal assumptions and moral intuitions would have adverse effects.

One of the respondents gave the following explanation to support the discussion above:

*“Yes. Suppose the people’s intentions are all concerned with self-benefits. In that case, they will be easily motivated by self-benefit to conduct unethical behaviors, especially in advantageous managerial positions.”*

In the interviews, only 52% of the respondents agreed that normative judgment forces corporate decision-makers to be unethical under the circumstances. Normative judgment theory is subject to highly controversial and massive criticisms. This theory suggested that human decision-making shall not solely be based on social norms of right or wrong but also consequential analysis and maximizing utility for the greatest number. In other words, whether the action is ethical should depend on the effects or consequences. If the action promotes maximum utilities and minimum prejudice for human beings, such action will be considered ethical, although it is unethical in the social norms of justice.

Nevertheless, 48% of the respondents disagreed with the normative judgment assumption. In their view, both shareholders and stakeholders hold equal rights. As the corporate decision-makers, their decision shall be fair, equitable, and ethical to all related parties. It is completely wrong and unethical to scarify certain party interests to promote another party’s interest.

The following quotation highlights the discussions above:

*“No. It is completely wrong that the management uses the excuse for majority interests to conduct unethically toward the minority. They shall not hide behind this clause which they have the same fiduciary duty toward either majority or minority.”*

All the respondents agreed that neuro-ethics is sufficient to explain corporate ethical decision-making. First, neuro-ethics significantly explains how emotions, personal moral values, assumptions, and moral intuitions impact human ethical judgments and behaviors. Second, to some extent, neuro-ethics draws out some fundamental perspectives on corporate unethical behaviors and provides competitive discussions on human moral judgments and behaviors.

### Human Nature of the Conflict of Interest and Self-Benefit Toward Unethical Behaviors

Almost all the respondents agreed that the human nature of conflict interests and self-interests are the fundamental drivers for unethical behaviors. This is once again is related to humans’ naturally selfish and greedy assumptions. Conflict of interest is a common problem in the contemporary business world and generally occurs when decision-makers (direct or indirect) clash between personal interests and fiduciary duties. Such a clash in interests could happen when transaction partners are decision-makers familiar with or have financial interests such as shares, commissions, or shares in profits with the transaction partners. We will lose our fairness in decision-making when faced with a conflict of interest, because our benefit is prioritized over social interests. If we need to choose between social or self-interests, we will be inclined toward our self-interests. Therefore, we would never sacrifice our interests.

One of the respondents provides the following scenario to support the discussion above:

*“For example, the company is now choosing suppliers. Both A and B suppliers offer similar products. Supplier A offers a slightly cheaper price than supplier B, but I could receive commissions from supplier B; I will go for B.”*

The scenario above explained what option we would adopt when facing a conflict of interests. However, the findings here slightly contrast with the social norm of fairness assumption discussed before. In the previous session, 20% of the respondents stated that there are some things beyond self-benefits and interests, like moral values. They will not feel motivated to conduct some things that are contradictory to their values and beliefs.

Nevertheless, all the respondents changed their minds if they could obtain the desired benefits from their formal authorities. Therefore, when people have formal authorities and legal positions to pursue interests, it is very likely that they will be motivated to misuse their formal authorities to obtain such interests. For example, one respondent has claimed that:

*“Yes. It is because the human will not be motivated to engage with the exchange without personal benefit. It will be perfect when the company and I could earn something extra from the deal.”*

Furthermore, the same discussions could also be applied when evaluating decision alternatives. We would not select alternatives that will damage our interests under normal circumstances. Humans tend to think about themselves before others. We would not reject any alternatives that could contribute positively. Therefore, we will naturally prefer options that could contribute positively to us. In other words, there is always a purpose behind every decision; humans could tolerate a decision if it does not benefit them but will not accept a decision that would damage their interests.

One of the respondents made the following statement:

*“Yes. Whatever decisions we make, it should not damage our interests. It is fine that we do not gain from the decision, but I definitely will not make the decision that would damage my interest even though it will benefit the company.”*

On the other hand, almost all of the respondents agreed that humans would be biased in making judgments when transaction parties are familiar with them. Due to their close relationship, we would adopt different sets of standards or criteria to evaluate the judgment options. Instead of considering the company and principal’s interests, their interests will always be our priority. Therefore, we will be compromised with our evaluation standards and show favoritism to the party. Furthermore, we always want to maintain good relationships with people familiar with us, so this personal feeling will distort our rationality in decision-making. Specifically, we will be afraid to lose such good relationships with them if we are not considerate and do not favor them, mainly when we are in favorable positions and have the formal authority to do that.

Regarding the discussion above, one of the respondents illustrated:

*“Yes. Since we are familiar with the transaction party, of course, we will be biased. We will be biased to ensure they can gain favorable returns from the transaction although their performances do not comply with company policies.”*

Apart from what was mentioned above, one respondent put forward the utility maximization assumption. This respondent suggested that there was no conflict between taking care of the company and the familiar party’s interests.

However, there is one respondent who disagreed with the discussions above. She argued that personal relationship should not be the basis of consideration. She stated that as corporate decision-makers, they have the fiduciary duty to act in the company and shareholders’ best interests, so they cannot be biased when making business decisions, and that all evaluations must be based on the company and shareholders’ best interests, not personal relationships.

From the financial incentive perspective, all the respondents agreed that financial incentives are the main driver that motivates managers to breach fiduciary relationships and conduct unethical behaviors. This is once again is related to the human nature of self-benefit and greed. Under the same concept, financial incentives (money or wealth) are common interests that we strive for in our life. Money is everything in the world; without money, we could not even survive in this world. In addition, human yearnings for a noble life and desires for luxury substances further enlarge human desire for money and wealth. Therefore, financial incentives can be the root of all evil behaviors.

When the agent-principal relationship comes into the picture, financial incentives are the underlying drivers that commonly motivate managers to commit a misconduct. For instance, when managers’ remunerations are tightly tied with company performance and the company is not performing well, the managers might be motivated to engage in fraudulent reporting to deceive principals into securing their well-paid remunerations. Furthermore, extrinsic rewards like salary and bonuses are good alternatives to motivate managers. However, if extrinsic rewards are not attractive enough or do not reach the so-called “justice level,” managers may be motivated to conduct something to get back what they think they deserve to receive.

The following quotations highlight some of the discussions above:

*“Yes, because money is everything in the world. If you don’t have money, you cannot do anything.”*

*“Yes, money will always help humans to satisfy what they want and need and therefore will do whatever even if there is an unethical situation.”*

*“Yes. When the management compensations are tightly related to the company performances, the unethical behaviors may occur. For example, cheating to overstate the performance.”*

Nevertheless, it is surprising that approximately 76% of the respondents agreed with the Etzioni (1988) argument that some people would behave ethically and not easily be influenced by desired rewards and interests. This finding was somewhat in contrast with the previous discussions. In their views, it is undeniable that humans are inherently greedy and willing to conduct whatever to pursue self-interests. Besides that, many of the respondents highlighted personal moral values. Moral values teach how to justify right and wrong. However, 24% of the respondents held on to their previous assumptions. They stated that humans would not behave ethically and with integrity when decisions would impact their interests.

As one of the respondents claimed:

*“I don’t think we will still act ethically when self-interest is involved.”*

## Discussion and Conclusion

Is an effective corporate governance structure important? Does neuro-governance provide significant explanations and examinations of how humans think, perceive, judge, and behave? Is truly understanding human behaviors and decision-making critical to mitigate corporate unethical behaviors and misconducts and enhance corporate governance functions? Through this research, we intend to answer the questions above.

At this point, it is evident that having an effective corporate governance structure is critical to safeguarding shareholders’ and stakeholders’ interests. Companies are faced with various inherent and systematic risks such as market competitions and economic conditions. Therefore, well-controlled internal control structures, optimized risk management, and independent auditing functions to optimize controllable and unsystematic risks have now come to vigorous attention.

However, past corporate scandals such as Enron and WorldCom demonstrated that ethical dilemmas, coupled with ineffective internal control structures, failure of audit functions, and defective board structures were the main contributions of these corporate failures, which had the thrust of human nature of greed, self-interest, and desire as well as conflict of interests. From this study, the social norm of fairness, to some extent, showed that the human nature of greed and selfishness would motivate corporate decision-makers to engage in any exchange that could benefit themselves, although it is unethical and illegal. Second, neuroeconomics revealed that scarcity of economic resources, level of risks and uncertainties, and expected rewards could be factors that motivate managers to conduct unethical behaviors, especially when their remunerations are tightly linked to company performances. Third, neuro-ethics shows that managers who lack moral values, have unstable emotions, and possess negative moral intuitions or personal assumptions could be more likely to pursue their interests at the cost of others. Lastly, neuro-governance also proves that self-benefits and financial incentives will usually be the priority. They would be motivated to conduct everything (both ethical and non-ethnic actions) to secure or pursue them.

On the other hand, neuro-governance also provides some views for enhancing corporate governance practices and business judgments, which concurs with previous studies. First, a high level of corporate disclosures could enhance corporate transparency and accountability, so companies shall maintain high commitments to disclose more information than the statutory. Disclosures shall also comply with existing reporting frameworks and standards. Second, past accounting records and accounting principles provide a comparison basis for decision-makers to evaluate performances and future improvements. Third, conflicts of interests may result in corporate decision-makers being biased in decision-making. Companies must have a code of conduct and policies for segregation of duties to eliminate conflict-of-interest occurrences. Lastly, although an intrinsic reward is a good alternative, companies shall also align the extrinsic reward with a sense of achievement and empowerment to motivate good performances.

Although we successfully overcame both the Asian Financial Crisis and Global Financial Crisis, the human nature of greed, self-interest persuasion, and financial temptations always motivates unethical managers to pursue loopholes or opportunities to advance their interests. With such understanding, at least we can know the rationale behind the unethical behaviors and act appropriately in situations. A study was conducted on the “current corporate governance among practitioners in Malaysian public listed companies.” Hence, neurological studies on governance have a string linkage to discover trends of decision making and its consequences in the corporate world.

Hence, this study strengthens the previous study globally and lays the foundation for how human brain activates in the context of corporate governance. It explores the influence of neuro-governance, neuro-ethics, neuro-accounting, neuroeconomics, and human nature in the corporate arena. This study concretizes the connection among the brain, emotions, past experiences, belief systems, and decision-making knowledge. It extends the theoretical contribution by addressing the relationship of neuro accounting, neuro-ethics, neuroeconomics, and human nature with corporate governance. The human brain’s condition with its environment plays a significant role in decision-making processes within the organization (Thagard, 2007).

### Study Limitations

The limitations of this study are as follow; (1) the data sample is small, perhaps a larger sample size could bring about a different result; (2) this research is purely conducted in Malaysia and within the Malaysian context; hence, international diversity in its data collection is absent; (3) a qualitative approach was undertaken in this study, perhaps a mixed method could bring greater robustness to this study.

### Implications for Future Research

Although this research covered fundamental frameworks to examine the neuro-governance views of corporate governance, few neuro-governance sub-disciplines were not included in this research, like neuro-leadership and neuro-philosophy. Neuro-leadership is an increasingly important discipline that studies leadership by combining neuroscience and central leadership elements like self-awareness, awareness of others, insight, and influencing others. Neuro-philosophy links neuroscience with that philosophical paradigm. Furthermore, there was also a lack of neuro-governance theory applications in the relevant case discussions. Therefore, future researchers may integrate neuro-governance theories into real case discussions. The researchers also agree that neuro-governance has a vast untapped area of research that is yet to be explored.

## Data Availability Statement

The raw data supporting the conclusions of this article will be made available by the authors, without undue reservation.

## Ethics Statement

Ethical review and approval was not required for the study on human participants in accordance with the local legislation and institutional requirements. The patients/participants provided their written informed consent to participate in this study.

## Author Contributions

All authors listed have made a substantial, direct, and intellectual contribution to the work, and approved it for publication.

## Conflict of Interest

The authors declare that the research was conducted in the absence of any commercial or financial relationships that could be construed as a potential conflict of interest.

## Publisher’s Note

All claims expressed in this article are solely those of the authors and do not necessarily represent those of their affiliated organizations, or those of the publisher, the editors and the reviewers. Any product that may be evaluated in this article, or claim that may be made by its manufacturer, is not guaranteed or endorsed by the publisher.
